# Multifunctional Gd³⁺- doped tellurite glass for photonic, radiation shielding, and dosimetric applications

**DOI:** 10.1186/s13065-026-01886-y

**Published:** 2026-07-28

**Authors:** T. Sivakumar, P. Vinothkumar, Manikandan Ayyar, Abdullatif Hakami, G. Lalitha, D. Shanmugapriya, Mohamed Yusuf

**Affiliations:** 1 Department of Physics, Saveetha Engineering College (Autonomous), Thandalam, Chennai, 602 105 India; 2 Centre for Material Chemistry, Karpagam Academy of Higher Education, Coimbatore, 641021 Tamil Nadu India; 3 Department of Chemistry, Karpagam Academy of Higher Education, Coimbatore, 641021 Tamil Nadu India; 4 Department of Electrical Engineering, College of Engineering, University of Bisha, P.O. Box 551, Bisha, 61922 Saudi Arabia; 5 International Center of Nanotechnology and Functional Materials, Instituto de Alta Investigación, Universidad de Tarapacá, Arica, 1000000 Chile; 6 Vicerrectoría de Investigación y Postgrado, Universidad de La Serena, Código, 1700000 Chile; 7 Department of Peace and Development Studies, Njala University, Bo Campus –18, Bo, Sierra Leone

**Keywords:** Gd³⁺-doped tellurite glass, Photoluminescence (PL), Radiation shielding, Thermoluminescence dosimetry, Electron paramagnetic resonance (EPR)

## Abstract

A novel Gd³⁺-activated tellurite glass with the composition 65TeO₂–15MgO–9SrO–5ZnO–5Ta₂O₅–1Gd₂O₃ (TeMgSrTa³⁺) was developed through the conventional melt-quenching route to investigate its combined optical, luminescent, electron paramagnetic resonance, thermoluminescence, and radiation shielding characteristics within a single glass matrix. The novelty of this work lies in the incorporation of Ta₂O₅ and Gd³⁺ into the TeO₂–MgO–SrO–ZnO glass network, enabling the simultaneous realization of ultraviolet photoluminescence, favorable dosimetric response, and efficient photon and neutron attenuation, which has not been comprehensively reported for this glass composition. X-ray diffraction confirmed the formation of a completely amorphous structure, while FTIR analysis identified TeO₄ and TeO₃ structural units responsible for network modification. UV–Visible spectroscopy revealed excellent optical transparency, and the characteristic absorption bands observed at 209, 247, and 347 nm were assigned to the 4f–5d transitions of Gd³⁺ ions. Under ultraviolet excitation, the glass exhibited a strong emission centered at 313 nm, originating from the ⁶P₇/₂→⁸S₇/₂ transition of Gd³⁺, with chromaticity coordinates of x = 0.1487 and y = 0.0468, demonstrating its suitability for ultraviolet photonic applications. Comprehensive gamma-ray shielding analysis showed close agreement between experimental and theoretical attenuation parameters, including the mass attenuation coefficient, linear attenuation coefficient, half-value layer, tenth-value layer, and mean free path across a broad photon energy range. The measured fast neutron effective removal cross-section further confirmed the material’s capability for neutron shielding. Thermoluminescence investigations produced well-defined glow peaks with favorable kinetic parameters, indicating promising dosimetric performance. Electron paramagnetic resonance analysis verified the valence state and local coordination environment of Gd³⁺ ions, providing insight into the ligand field surrounding the activator. The synergistic combination of structural stability, ultraviolet luminescence, radiation attenuation efficiency, and dosimetric response demonstrates that the proposed TeMgSrTa³⁺ glass is a promising multifunctional material for advanced photonic devices, radiation protection systems, and radiation sensing technologies.

## Introduction

Radioactive radiation originates from both natural and artificial sources, including cosmic rays, nuclear reactors, nuclear weapons, and various industrial and chemical processes. These sources emit different types of ionizing radiation, such as gamma (γ) rays, X-rays, charged particles, and neutrons. Among them, γ-rays are highly energetic electromagnetic radiation that possess neither mass nor electric charge, allowing them to penetrate materials deeply and travel over long distances. Due to their high penetrating ability, prolonged exposure to γ-radiation can cause serious biological damage, including DNA damage, disruption of blood cell production, an increased risk of cancer, and, at high radiation doses, irreversible tissue damage or death [1–3]. Considering these advantages and the increasing demand for advanced shielding materials, significant research efforts have been directed toward the development of alternative gamma-radiation shielding materials that can overcome the limitations of conventional shields. In particular, glass-based systems have emerged as promising candidates due to their ability to combine effective radiation attenuation with favorable optical, structural, and mechanical properties [4, 5]. Compared with conventional radiation shielding materials such as lead, concrete, alloys, and natural rocks, glass has attracted considerable attention owing to its unique combination of functional properties. Traditional shielding materials are generally opaque, heavy, difficult to transport, and unsuitable for applications requiring optical transparency. In contrast, glass is an amorphous material that offers high transparency in the visible region, excellent chemical and mechanical stability, good environmental durability, ease of fabrication, and relatively low production cost. These advantages make glass a promising candidate for radiation shielding applications where both effective protection and optical functionality are required. Tellurite glasses have attracted considerable attention because of their low phonon energy, wide optical transmission window, high refractive index, and excellent rare-earth ion solubility, making them promising candidates for photonic and radiation-related applications. Previous studies have primarily focused on either luminescence enhancement or radiation shielding performance, with only limited attention given to integrating these functionalities within a single glass composition. Furthermore, the combined influence of Ta₂O₅ as a network modifier and Gd³⁺ as an activator on the structural, optical, dosimetric, and shielding characteristics of tellurite glasses has not been systematically investigated. The absence of such comprehensive studies restricts the development of multifunctional materials capable of serving both photonic and radiation protection technologies [6–8]. Additionally, tellurite glasses can be employed in a diversity of uses, including solar cell devices, solid – state lasers, sensors, optical fibres, and optoelectronics [9].

Germanate, tellurite, and germanate–tellurite glass systems have attracted considerable research interest owing to their distinctive physicochemical properties, including high dielectric constants, relatively low melting temperatures, excellent infrared transparency, and wide thermal stability ranges. These characteristics make them promising candidates for a variety of optical, photonic, and functional applications [10, 11]. Furthermore, the electrical, thermal, optical, and radiation protecting properties of tellurite (TeO_2_) glass structures are enhanced by the accumulation of RE (rare earth) oxides like Er_2_O_3_, La_2_O_3_, Sm_2_O_3_, Pr_6_O_11_, and Nb_2_O_5_, or transition metal oxides (TMOs) like SrO, CaO, ZnO, WO_3_, Na_2_O, MgO, TaO_5_ and CoO_3_, [12, 13]. The gamma/neutron blocking characteristics of 21 – tellurite glasses with various shapes that contained TMOs and REs were reported and they stated that the protecting qualities of these glasses are higher – ranking materials [14].

Previous studies have examined the radiation shielding performance of TeO₂-based glasses containing rare-earth oxides such as Nd₂O₃ and Sm₂O₃. The reported results demonstrated that the incorporation of these rare-earth ions enhances the gamma-ray attenuation capability of the glass matrix. Likewise, transparent tellurite glasses doped with Pr₆O₁₁, Sm₂O₃, Ho₂O₃, Tm₂O₃, and NdCl₃ have been investigated for neutron and gamma-ray shielding applications. These studies consistently confirmed that rare-earth incorporation improves the radiation shielding effectiveness of tellurite glass systems [15, 16].

The incorporation of rare-earth oxides into tellurite-based glass systems has been shown to enhance their radiation shielding performance. For example, the addition of Sm₂O₃ to TeO₂–ZnO glasses improves gamma-ray attenuation as well as alpha and neutron shielding capabilities. Similarly, tellurite-, borate-, and tellurite–borate-based glasses containing rare-earth oxides such as Pr₆O₁₁, Dy₂O₃, NdCl₃, Y₂O₃, and Gd₂O₃ have demonstrated superior radiation shielding efficiency compared with conventional shielding materials, including commercial glass and ordinary concrete. These findings highlight the potential of rare-earth-doped tellurite glasses for advanced radiation protection applications [17, 18]. Furthermore, the incorporation of ZnO as a network modifier significantly enhances the properties of tellurite glasses by improving glass-forming ability, lowering the melting temperature, suppressing crystallization, increasing chemical durability, and enhancing the refractive index. These improvements contribute to the excellent optical and functional performance of tellurite-based glass systems. In addition, Gd₂O₃-doped glass matrices have attracted considerable attention because Gd³⁺ ions exhibit highly efficient energy transfer to activator ions. This characteristic enhances their luminescence efficiency, making these glasses promising materials for scintillation applications, radiation detectors, and high-energy particle detection systems [19, 20].

Based on their unique structural characteristics, the TeO₂–MgO–SrO–ZnO–Ta₂O₅–Gd₂O₃ glass system is expected to exhibit promising radiation attenuation performance, particularly against high-energy X-rays and gamma rays. To the best of our knowledge, the radiation shielding characteristics of this glass composition have not yet been systematically investigated.

The selection of the TeO₂–MgO–SrO–ZnO–Ta₂O₅–Gd₂O₃ glass system is based on the complementary contributions of its constituent oxides. TeO₂ serves as the primary glass former, offering excellent optical transparency and superior glass-forming characteristics. The incorporation of MgO and SrO enhances the structural integrity, mechanical strength, and thermal stability of the glass network. ZnO acts as a network modifier, improving chemical durability, optical nonlinearity, and the connectivity of the glass structure. Ta₂O₅, with its high atomic number and density, is expected to enhance the radiation attenuation capability of the glass. Furthermore, Gd₂O₃ is introduced because Gd³⁺ ions possess remarkable paramagnetic properties, a high neutron capture cross-section, and characteristic luminescence, enabling the glass system to exhibit multifunctional performance suitable for photonic, radiation shielding, and dosimetric applications.

Therefore, the present study aims to evaluate the structural, optical, thermoluminescence dosimetric, electron paramagnetic resonance (EPR), and radiation shielding properties of Gd³⁺-doped tellurite glass. The shielding performance against gamma rays, neutrons, protons, and alpha particles is examined using both experimental measurements and theoretical calculations to assess its potential for radiation protection applications.

The radiation shielding performance of the prepared glass was comprehensively evaluated by determining the mass attenuation coefficient (MAC, µ/ρ) over a wide photon energy range of 0.001–15 MeV. Based on the calculated MAC values, the corresponding linear attenuation coefficient (LAC), half-value layer (HVL), tenth-value layer (TVL), and mean free path (MFP) were determined to assess the photon attenuation capability of the glass. In addition, the fast neutron removal cross-section was estimated to investigate its effectiveness against neutron radiation. The thermoluminescence (TL) characteristics were examined to evaluate the suitability of the glass for radiation dosimetry by determining key kinetic parameters, including the order of kinetics (b′), activation energy (E′), and frequency factor (s), which describe the trapping and recombination mechanisms. Furthermore, electron paramagnetic resonance (EPR) spectroscopy was employed to investigate the oxidation state, coordination environment, and local ligand field symmetry of the Gd³⁺ ions incorporated into the TeMgSrTa glass matrix, thereby providing valuable information on the relationship between the local structural environment and the observed optical and dosimetric properties. The present work introduces a Gd³⁺-activated prepared glass system and evaluates its structural, optical, photoluminescence, electron paramagnetic resonance, thermoluminescence, and radiation shielding properties within a unified framework. This integrated investigation establishes the relationship between glass structure and multifunctional performance, providing valuable insight into the design of advanced materials for ultraviolet photonics, radiation dosimetry, and nuclear shielding applications.

## Materials, synthesis and simulation methods

### Preparation of glass samples

**Fig. 1:**
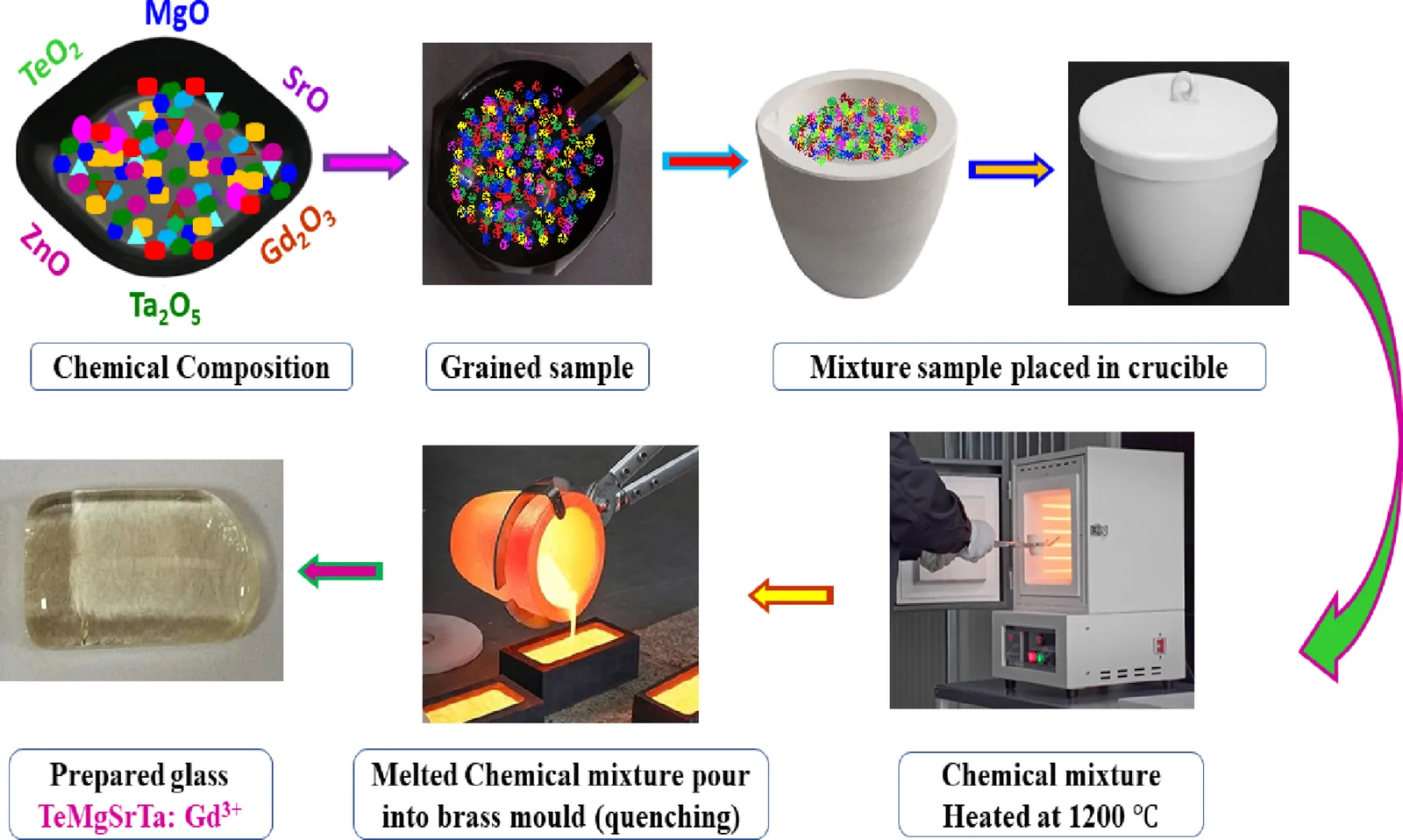
Chemical reaction and synthesis procedure of TeMgSrTa: Gd^3+^ glass

The Gd³⁺-doped tellurite glass with the molar composition 65TeO₂–15MgO–9SrO–5ZnO–5Ta₂O₅–1Gd₂O₃ (denoted as TeMgSrTa³⁺) was synthesized using the conventional melt-quenching technique. High-purity (99.9%, AR grade) TeO₂, MgO, SrO, ZnO, Ta₂O₅, and Gd₂O₃ (Sigma-Aldrich) were accurately weighed according to the desired molar composition. The precursor powders were thoroughly mixed in an agate mortar until a homogeneous fine powder was obtained. The mixed batch was transferred to a platinum crucible and melted in an electrically controlled furnace at 900 °C for 40 min, with intermittent stirring to promote complete homogenization and eliminate trapped air bubbles. The molten glass was then poured onto a preheated metal mould and pressed to produce transparent glass specimens. Subsequently, the samples were annealed at 350 °C for 2 h to relieve residual thermal stresses and then cooled gradually to room temperature. This synthesis route ensured the successful preparation of a homogeneous tellurite-based glass system through the controlled incorporation of network-forming and network-modifying oxides. A schematic illustration of the synthesis procedure and the corresponding reaction mechanism is presented in Fig. 1 [21]. The detailed chemical reaction mechanism and synthesis procedure is picturized in Fig. 1.

### The shielding and dosimetric calculations by Phy-X/PSD

A thorough understanding of the interaction between gamma photons and glass materials is essential for evaluating their suitability as radiation shielding media in advanced technological applications. This evaluation is primarily based on determining the photon attenuation characteristics of the material, which describe its ability to reduce the intensity of incident gamma radiation. Parameters such as the mass attenuation coefficient, linear attenuation coefficient, half-value layer, tenth-value layer, and mean free path are widely used to assess shielding effectiveness and are fundamental in the design of radiation protection systems and dosimetric applications [22]. The radiation shielding performance of a material is commonly evaluated using several photon attenuation parameters, including the mass attenuation coefficient (MAC), linear attenuation coefficient (LAC), half-value layer (HVL), tenth-value layer (TVL), mean free path (MFP), effective electron density, and buildup factors. Accurate determination of these parameters over a wide photon energy range is essential for assessing the shielding efficiency of candidate materials. Such evaluations enable reliable comparison among different glass systems and facilitate the selection and design of advanced radiation shielding materials for practical nuclear, medical, and industrial applications [23]. For our study to accurately calculate these shielding and dosimetry-related parameters, we used an intuitive web program called Phy-X/PSD (Photon protecting and Dosimetry). For additional details on the online tool could be originate in the webpage of https://phy-x.net/PSD [5]. The photon attenuation parameters were calculated using specialized radiation shielding software capable of evaluating multiple shielding and dosimetric quantities over a wide photon energy range. By entering the material density, chemical composition, and photon energy, the software accurately determines the relevant attenuation parameters. Among these, the mass attenuation coefficient (MAC, µ/ρ) is the primary parameter used to assess the photon attenuation capability of the glass, as it describes the probability of photon interaction with the constituent atoms. Furthermore, the MAC serves as the basis for calculating other important shielding parameters, including the linear attenuation coefficient (LAC, µ), half-value layer (HVL), tenth-value layer (TVL), mean free path (MFP), and fast neutron removal cross-section (ΣR), which collectively provide a comprehensive evaluation of the radiation shielding performance of the material.

#### Theoretical analysis

Shielding attenuation coefficients: Consider a monoenergetic photon beam with an initial intensity incident on an absorbing material of thickness (x). As the photons propagate through the medium, their intensity decreases exponentially according to the Beer–Lambert law, which can be expressed mathematically as follows:.

1 $$\:{\upmu\:}=\:-\:\frac{\mathrm{I}\mathrm{n}\:\frac{\mathrm{I}}{{\mathrm{I}}_{\mathrm{o}}}}{\mathrm{X}}$$

where, µ is the LAC (cm ^–1^), I and Io represents attenuated and un-attenuated radiation intensities. The MAC – mass attenuation coefficient (µ/ρ), in contrast, is the interaction possibilities of photons with the barrier and it is given as follows for each chemical combination or mixture of elements by the relation [24]:

2 $$\:\frac{{\upmu\:}}{{\uprho\:}}=\:\sum\:{\mathrm{w}}_{\mathrm{i}}\:({\upmu\:}/{\uprho\:}{)}_{\mathrm{i}}\:\:\:\:\:\:\:\:\:\:\:\:\:\:\:\:\:\:\:\:\:\:\:\:\:\:\:\:\:\:\:\:\:\:\:\:\:\:\:\:\:\:\:\:\:\:\:\:\:\:\:\:\:\:\:\:\:\:\:\:\:\:\:\:\:\:\:\:\:\:\:\:\:\:\:\:\:\:\:\:\:\:\:\:\:\:\:\:\:\:\:\:\:\:\:\:\:\:\:\:\:\:\:\:\:\:\:\:\:\:\:$$

where ρ – represent the density of the sample, (µ/ρ)_i_ - be the MAC of individual element of each component and w_i_ – indicates fractional weight.

The MFP, which is based on µ, it is estimated by increasing the consistent of µ/ρ and density, indicates the regular distance a radiation can travel through the barrier before encountering an interaction. The equation of MFP is given below [25],

3 $$\:\mathrm{M}\mathrm{F}\mathrm{P}=\:\raisebox{1ex}{$1$}\!\left/\:\!\raisebox{-1ex}{${\upmu\:}$}\right.$$

The depth of the interaction target corresponding to attenuated intensity of 90% and 50% of the narrowest photon beam intensity is referred to as the TVL and HVL, respectively. The TVL and HVL ratios are inversely related to the required layer thickness of protecting material. It can be ascertained as follows [26].

5 $$\:\mathrm{T}\mathrm{V}\mathrm{L}=\:\raisebox{1ex}{$\mathrm{I}\mathrm{n}10$}\!\left/\:\!\raisebox{-1ex}{${\upmu\:}\:$}\right.\:\:\:\:\:\:\:\:\:\:\:\:\:\:\:\:\:\:\:\:\:\:\:\:\:\:\:\:\:\:\:\:\:\:\:\:\:\:\:\:\:\:\:\:\:\:\:\:\:\:\:\:\:\:\:\:\:\:\:\:\:\:\:\:\:\:\:\:\:\:\:\:\:\:\:\:\:\:\:\:\:\:\:\:\:\:\:\:\:\:\:\:\:\:\:\:\:\:\:\:\:\:\:\:\:\:\:\:\:\:\:\:\:\:\:\:$$

6 $$\:\mathrm{H}\mathrm{V}\mathrm{L}=\:\raisebox{1ex}{$\mathrm{I}\mathrm{n}2$}\!\left/\:\!\raisebox{-1ex}{${\upmu\:}\:$}\right.\:\:\:\:\:\:\:\:\:\:\:\:\:\:\:\:\:\:\:\:\:\:\:\:\:\:\:\:\:\:\:\:\:\:\:\:\:\:\:\:\:\:\:\:\:\:\:\:\:\:\:\:\:\:\:\:\:\:\:\:\:\:\:\:\:\:\:\:\:\:\:\:\:\:\:\:\:\:\:\:\:\:\:\:\:\:\:\:\:\:\:\:\:\:\:$$

FNRCS: The prospect of neutrons passing through a substance without interacting is shown by the effective Σ_R_. Any absorber’s Σ_R_ can be determined to the following relation [27],

7 $$\:\:\sum\:_{i}R=\:\sum\:_{i}{w}_{i\:}\:(\sum\:_{i}{R/\rho\:)}_{i}$$

8 $$\:{\Sigma\:}\mathrm{R}\:=\sum\:_{i}{\rho\:}_{i}\:{\left(\sum\:R/\rho\:\right)}_{i}$$

where $\:{\left(\sum\:R/\rho\:\right)}_{i}$ represents i^th^ element RCS and w_i_ denotes partial density of element.

Dosimetric parameters: The samples were exposed to a 10 kGy radiation dose, and an Intech TLD (I-1501 dc) reader was used to record their TL glow curves. The glass was pre-hardened to eliminate excess optical signs, and the thermoluminescence glow peaks were recorded from ambient temperature to 400 °C at a rate of heating 5 °C/s. The TL glow peak reveal the light emitted from different trapping stages within the optical energy band gap of the host glass material.

The dosimetric uses of the TL glass materials are calculated by kinetic factors like kinetic order (bʹ), factor of frequency (s), and activation energy (Eʹ) or trap depth. This study employs Chen’s glow peak shape approach to assess these parameters from the light curve. Here, T_1_ and T_2_ indicates the temperatures at the full width and half maximum of the glow curve peak, respectively, while T_m_ represents the temperature at the extreme intensity peak. We must compute the complete width at the maximum of the peak (ω), the half width at high temperature (δ), and the half width at low temperature (τ) in order to determine the kinetic parameters [28].

9 $$ \begin{gathered} \omega = {\text{ }}\left( {{\mathrm{T}}_{{\mathrm{2}}} - - {\text{ T}}_{{\mathrm{1}}} } \right); \hfill \\ \delta = {\text{ }}\left( {{\mathrm{T}}_{{\mathrm{2}}} - - {\text{ T}}_{{\mathrm{m}}} } \right);{\text{ }} \hfill \\ \tau = {\text{ }}\left( {{\mathrm{T}}_{{\mathrm{m}}} - - {\text{ T}}_{{\mathrm{1}}} } \right) \hfill \\ \end{gathered} $$

Kinetic parameters, including the geometric factor (µ_g_), activation energy (E_τ_ for the ascending portion of the light curve, E_δ_ for the descending portion, and E_ω_ for the entire glow curve), and factor of frequency (s), were computed utilizing Chen’s curve shape approach as expressed below,

10 $${\mu _{\mathrm{g}}}\,=\,\delta /\omega ={\text{ }}\left[ {\left( {{{\mathrm{T}}_{\mathrm{2}}}--{\text{ }}{{\mathrm{T}}_{\mathrm{m}}}} \right){\text{ }}/{\text{ }}\left( {{{\mathrm{T}}_{\mathrm{2}}}--{\text{ }}{{\mathrm{T}}_{\mathrm{1}}}} \right)} \right]$$

By applying, the peak shape method applicable for first-order kinetics, the activation energy (Eʹ_α_) of the trapping centre was estimated by the relation [29, 30].

The empirical formula,

11 $$ {\mathrm{E}}^{\prime } _{\alpha } = {\text{ }}\left\{ {{\mathrm{C}}^{\prime } _{\alpha } [{\mathrm{K}}_{{\mathrm{B}}} T_{m}^{2} /\alpha ]{\text{ }} - {\mathrm{b}}^{\prime } _{\alpha } \left( {{\mathrm{2T}}_{{\mathrm{m}}} } \right)} \right\} $$

where K_B_ denotes Boltzmann’s constant and T_m_ represents temperature of peak (K), Cʹ_α_, bʹ_α_ be the Chen’s constants of activation energy.

### Characterization analysis

The amorphous nature of the prepared glass was examined using a Bruker D8 powder X-ray diffractometer operated at a scanning rate of 2° min⁻¹ over a 2θ range of 5°–90°. Fourier transform infrared (FTIR) spectra were recorded using the KBr pellet technique with a PerkinElmer Lambda 35 spectrometer in the spectral range of 400–4000 cm⁻¹. Optical absorption measurements were carried out using a PerkinElmer LAMBDA 950 double-beam UV–Vis–NIR spectrophotometer. Photoluminescence (PL) spectra were recorded using a Horiba Jobin Yvon monochromator spectrometer, and the corresponding CIE 1931 chromaticity coordinates were determined from the PL emission spectra. For thermoluminescence (TL) analysis, the glass samples were irradiated with γ-rays from a Co-60 source at a dose of 10 kGy using a GC-5000 γ-ray irradiator (BRIT) operating at a dose rate of 40 Gy min⁻¹. The TL glow curves were recorded from room temperature to 300 °C at a heating rate of 5 °C s⁻¹ using an Intech Dosimeters TL analyzer. Electron paramagnetic resonance (EPR) measurements were performed at room temperature using a JEOL JES-FA200 EPR spectrometer.

## Result and discussion

### Structural analysis

#### Powder X-ray study

**Fig. 2:**
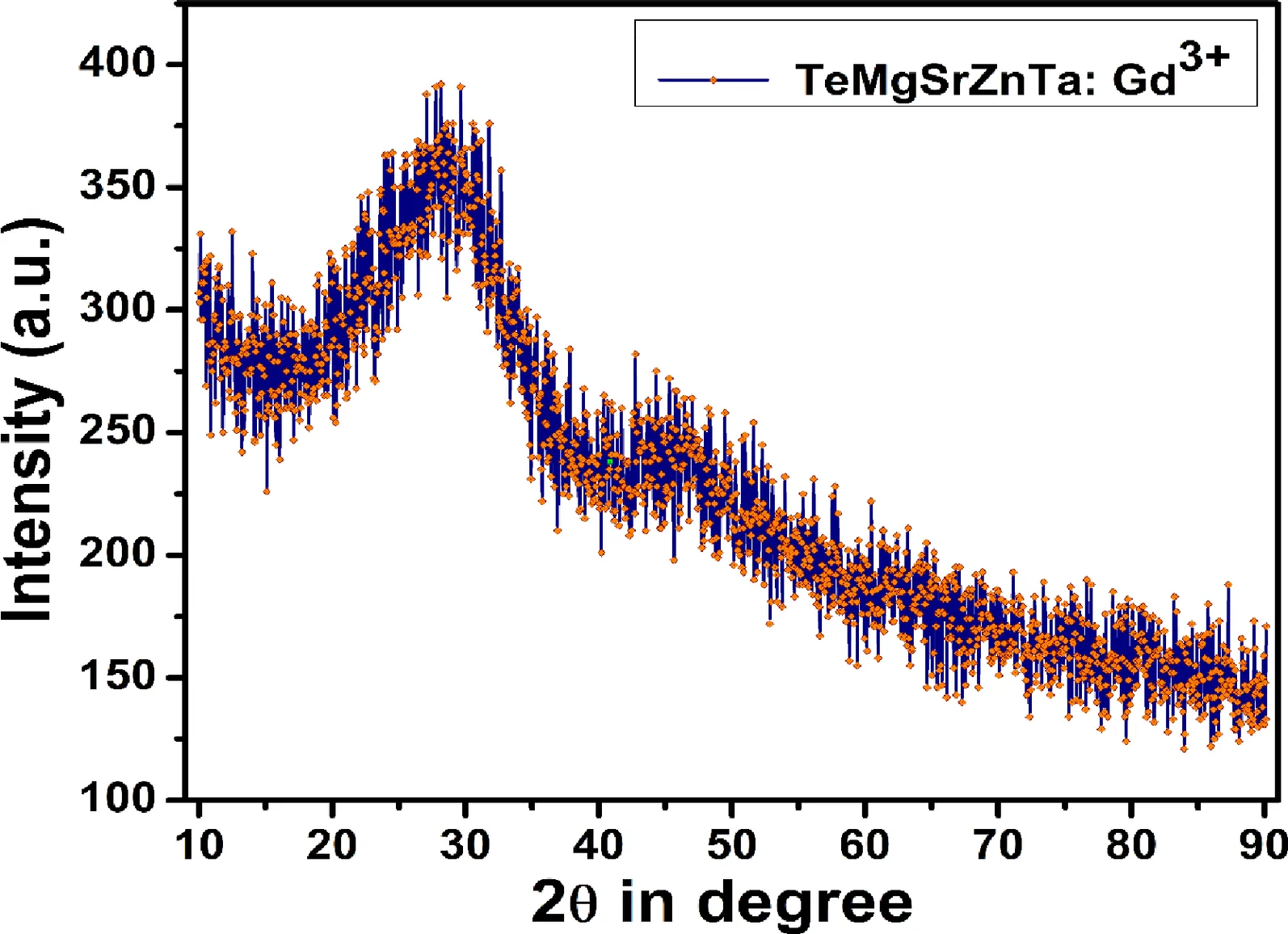
X-ray powder diffraction pattern of TeMgSrTa: Gd^3+^ glass

X-ray powder diffraction (XRD) is a widely used technique for evaluating the structural characteristics and confirming the amorphous nature of glass materials. Figure 2 presents the XRD pattern of the TeO₂–MgO–SrO–ZnO–Ta₂O₅–Gd₂O₃ (TeMgSrTa³⁺) glass system. A broad diffuse halo is observed in the 2θ range of 25°–35°, with no sharp diffraction peaks, confirming the absence of long-range crystalline order. This characteristic broad hump indicates the formation of a homogeneous amorphous glass network. The observed non-crystalline structure is consistent with the random arrangement of structural units in tellurite-based glasses and agrees well with previously reported studies on rare-earth-doped tellurite glass systems [31].

#### IR vibrational analysis

**Fig. 3:**
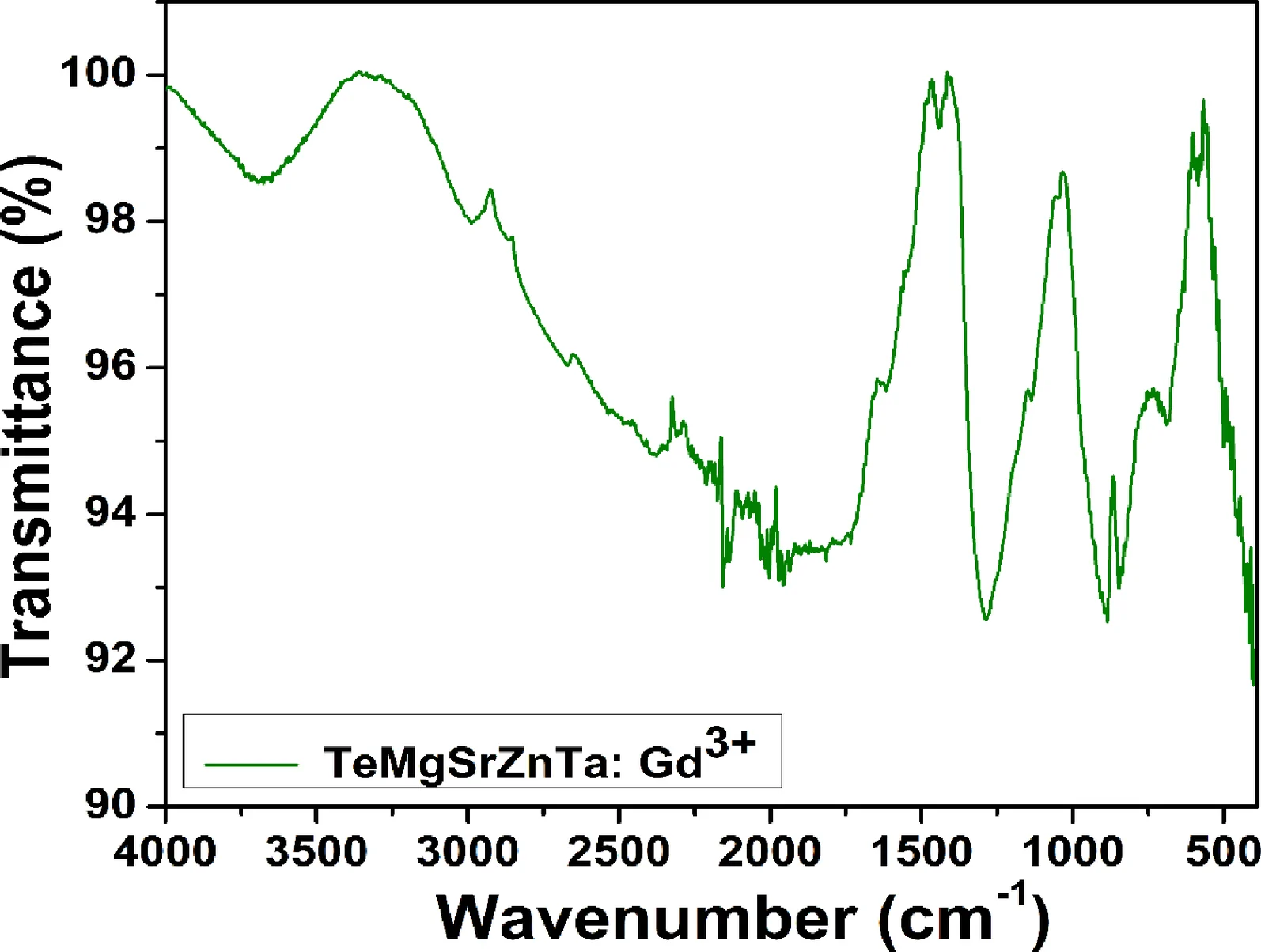
IR Vibrational spectroscopy graph of Gd^3+^ doped TeMgSrTa glass

Fourier transform infrared (FTIR) spectroscopy is an effective complementary technique for investigating the structural features of glass materials. The infrared absorption bands originate from changes in the dipole moment associated with the vibrational motions of chemical bonds within the glass network. Therefore, FTIR analysis provides valuable information on the local structural units and bonding environment. In the present study, FTIR spectroscopy was employed to identify the characteristic vibrational bands and structural groups present in the TeO₂–MgO–SrO–ZnO–Ta₂O₅–Gd₂O₃ (TeMgSrTa³⁺) glass system. Figure 3 illustrates the peaks appeared at 416–429 cm^− 1^ is ascribed to the symmetric mode of stretching vibrations for Zn – O in ZnO_4_. The bending mode vibrations of (Te – O – Te) linkages are ascribed to the peak at 451–480 cm^− 1^, which reduces in wavenumber when the glass network of TeO_2_ was supplemented with Gd_2_O_3_ [32]. The TeO_4_ – band’s stretching vibrations are noticed amid 570 and 582 cm^− 1^. The TeO_4_ –vibration of stretching band is observed between 655 and 678 cm^− 1^. The axially vibrational modes of symmetrical stretching (Te_ax_ – O) in the tellurite tetrahedral (TeO_4_) are accountable for the band at 620–650 cm^− 1^ [33, 34]. Te – O vibration in trigonal bi-pyramids, TeO_4_, is responsible for 600–642 cm^− 1^, while Te–O trigonal pyramids vibration, TeO_3_, is responsible for 680–700 cm^− 1^. The peak at 735–751 cm^− 1^ ascribed to the TeO_3_ stretching mode vibration [35]. There are two possible interpretations for the band at about 1134 and 1291 cm^− 1^. The bending vibration arises due to Te–O–Zn or Ta–O terminal group of stretching mode vibration [36, 37]. The peak occurred at about 570 cm^− 1^, as we have seen, described GdO_4_ and Gd–O–Gd bending modes [31].

#### Linear optical analysis

**Fig. 4:**
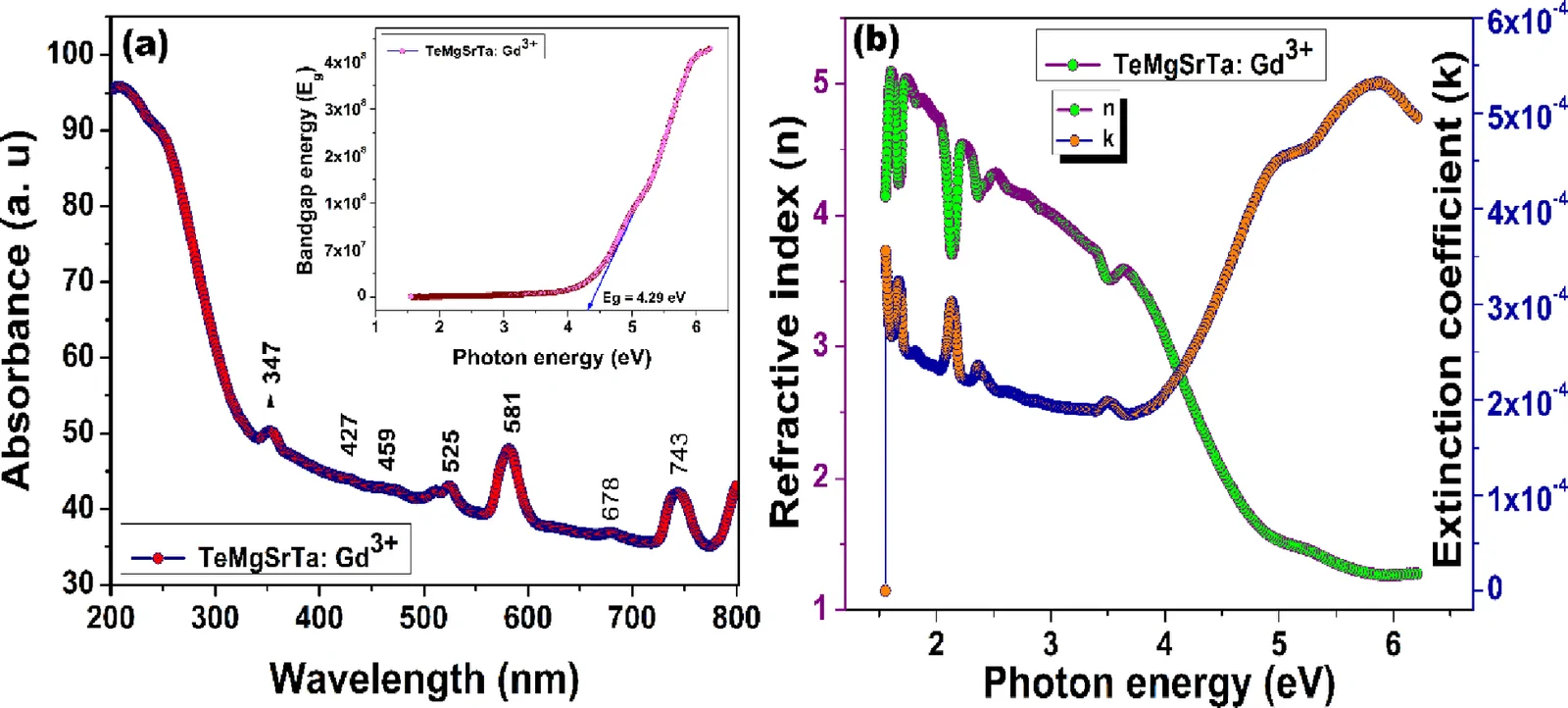
**a** Linear optical observation and Inset Tauc’s plot; **b** graphical representation of ‘n’ and ‘k’

The optical absorption characteristics of the Gd³⁺-doped tellurite glass were investigated using UV–Vis spectroscopy over the wavelength range of 200–800 nm, as shown in Fig. 4a. The absorption spectrum exhibits enhanced absorption in the ultraviolet region, which is attributed to the incorporation of Gd³⁺ ions into the tellurite glass matrix. The observed absorption bands originate from the characteristic electronic transitions of Gd³⁺ ions associated with their partially filled 4f electronic configuration. Rare-earth ions possess the general electronic configuration [Xe]4fⁿ5d⁰6s², where the partially occupied 4f shell gives rise to well-defined optical transitions, making them attractive activators for optical and photonic applications [38]. As a result, their optical transitions over glass generally resembles intra-f^N^ electric dipole (ED) energy transitions oddity, and their observable absorption peaks are found in the infrared (IR) to ultraviolet (UV) spectrum. UV absorption is created by the shift from the lower energy state 4f to the higher energy state 5 d, whereas visible to near-IR absorbance occurs during the shifts between f–f energy transitions [39, 40].

Figure 4a presents the UV–Vis–NIR absorption spectrum of the synthesized TeMgSrTa³⁺ glass. The spectrum exhibits distinct absorption bands centered at approximately 209, 247, and 347 nm, which are attributed to the 4f–5d electronic transitions of Gd³⁺ ions. These characteristic absorption bands confirm the successful incorporation of Gd³⁺ ions into the tellurite glass network without altering its amorphous nature. The observed ultraviolet absorption behavior is characteristic of Gd³⁺-activated glass systems and demonstrates the suitability of the prepared glass for ultraviolet photonic and optoelectronic applications [41]. As is well known, gadolinium ions consist of two oxidation states stably: Gd^3+^ and Gd^4+^, which have the electrical configurations f_1_ and f_0_, respectively. The permitted transition (f_1_→ d_1_) in the absorption of Gd^3+^ ions functions as wide and extensive absorption bands in a variety of solid environments [42]. It is highly dependent on the host composition and typically manifests in the UV region in the majority of oxides. However, the charge transfer characteristic of O_2_→ Gd^4+^ is what causes the absorption peaks connected to Gd^4+^, which usually appear at a longer wavelength than that of Gd^3+^ [43].

Herein the absorption spectrum of Gd^3+^ doped glass exhibits the significant absorption peaks at 347, 427, 459, 581, 678 and 743 nm due to the energy transitions spectrum of ^4^P_5/2_, ^4^G_11/2_, ^4^I_15/2_, ^6^H_13/2_, ^4^F_9/2_ and ^6^F_3/2_ Linear optical possessions of synthesised glass are studied using the UV-Vis spectrum. The optical bandgap energy (E_g_) from the linear optical absorption edge is determined using the Moot and Davis theoretical method. The optical band gap energy and associated parameters of the glasses under study are investigated using one of the fundamental optical parameters, α (optical absorption coefficient), which is derived from equation following relation α = 2.303 A_bs_/tʹ, where, A_bs_ be the optical absorption and tʹ stands thickness of the glass. The relation amid the optical absorption coefficient (α) and bandgap energy (E_g_) is given as follows [44],

12 $$(\alpha h\upsilon ){\text{ }}={\text{ }}B^{\prime}{\text{ }}{(h\upsilon -{\text{ }}{E_g})^n}$$

Where, Bʹ be the constant, hυ stands photon energy of incident beam and n be the electronic transition index which holds the direct and indirect permitted energy transition. Figure 4a – inset, reveals that the Tauc’s plots and the graph plot between the (αhυ)^1/2^ verses hυ, where *n* = 2, and ½. The value of bandgap energy (E_g_) found to be 4.29 eV for Gd^3+^ added TeMgSrTa glass. The obtained value of E_g_ is increased with the dopant Gd^3+^ added, which attributed to excess amount of non – bridging oxygens between the glass matrix [45]. Nevertheless, doping the glass with Gd_2_O_3_ resulted in a wider optical band gap, which decreased the quantity of NBOs. In this research on the optical characterisation of some boron and tellurium based glasses showed that doping with RE ions increased the optical band gap [46].

The refractive index (n) of the material is a crucial parameter in optical physics that characterizes the electronic polarization of ions within a material. It is pivotal in the advancement of spectrum dispersion instruments and optical communication. The n (refractive index) and k (extinction coefficient) has been estimated suing the following relation,

13 $$\:\:\:\:\:\:n=\:\frac{(1+R)}{(1-R)}\:\:\:{\left[\frac{4R}{{(1\:-\:R)}^{2}}\:-\:{k}^{2}\right]}^{1/2}$$

14 $$\:k\:=\:\left[\raisebox{1ex}{$\alpha\:\lambda\:$}\!\left/\:\!\raisebox{-1ex}{$4\pi\:$}\right.\right]$$

The n and k variations with incident beam wavelength are depicted in Fig. 4b. The calculated values of n and k found to be 1.61 and 4.62 × 10^− 4^ respectively. The obtained values of different linear optical parameters reflect on the application of recent borate and tellurite glasses in the field of photoelectric and optoelectronic devices.

#### Photoluminescence and CIE – 1931 study

**Fig. 5:**
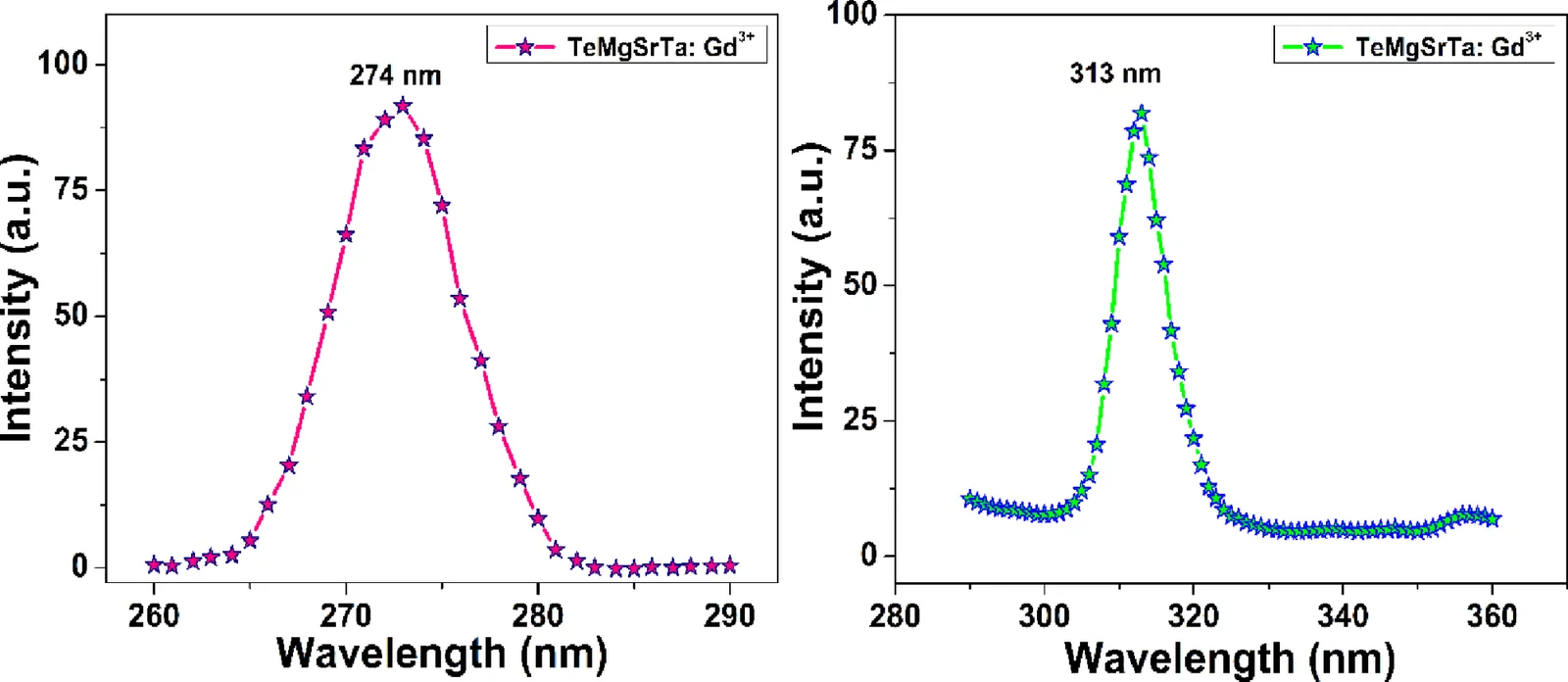
Excitation and emission spectra of TeMgSrTa: Gd^3+^ glass

Photoluminescence (PL) excitation and emission spectra of TeMgSrTa: Gd^3+^ glass material as illustrated in Fig. 5a, b. The presence of Gd^3+^ ions in Gd_2_O_3_ excitation energy transitions within Gd^3+^ doped glass materials was further analyzed to investigate the excitation spectra. There is only one excitation peak of Gd^3+^ ions dopant glass exhibits at 274 nm and the related energy transition ^8^S_7/2_ → ^6^I_9/2_ [47, 48]. Moreover, PL emission spectrum of TeMgSrTa: Gd^3+^ glass material was noticed by the excitation, as picturized in Fig. 5. Due to the Gd^3+^ ions dopant a permanent emission peak is occurred at 313 nm under the excited wavelength at 274 nm and it is corresponding to the energy transition ^6^P_7/2_ → ^8^S_7/2_ [49].

**Fig. 6:**
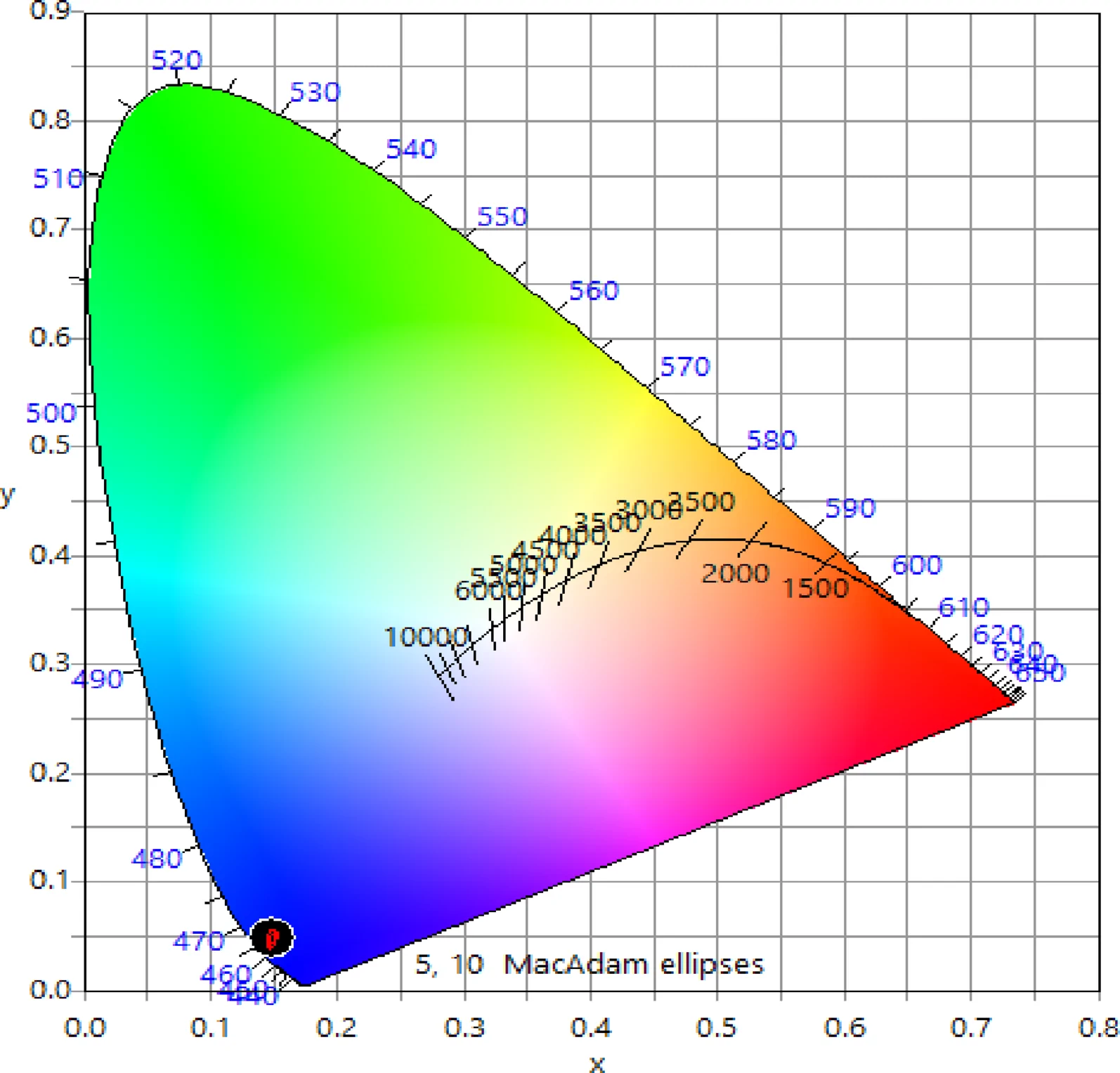
Colour chromaticity diagram of TeMgSrTa: Gd^3+^ glass

The visible light emission color that the human’s eye can perceive was measured using Commission international de I’Eclairage (CIE – 1931) coordinates. The sensitivity of the human visual system to different color wavelengths is taken into account while calculating the CIE, a typical for color perseverance [50]. As seen in Fig. 6, the measured color coordinate values for the Gd^3+^ glass lie inside the blue area of the CIE diagram. The quality of the light source is assessed using the correlated color temperature (CCT), which shows the temperature of the Plankain’s black body locus closest to the chromaticity diagram’s operative point and colour coordinates and the purity of the prepared glass was calculated [50, 51].

The emission characteristics of the TeMgSrTa³⁺ glass were further evaluated using the CIE 1931 chromaticity diagram, which was constructed from the photoluminescence emission spectrum. The calculated chromaticity coordinates are x = 0.1487 and y = 0.0468, with a corresponding color purity of 95.7%. These values are in close agreement with the standard blue emission region defined by the National Television System Committee (NTSC) color space, confirming the high color purity of the emitted light. The obtained chromaticity coordinates indicate that the prepared glass exhibits intense blue emission, demonstrating its potential for ultraviolet–blue photonic devices, solid-state lighting, and display applications. Furthermore, the emission color of tellurite glasses can be effectively tailored by selecting appropriate network modifiers, providing additional flexibility for the development of advanced luminescent materials [40]. Consequently, the produced glass exhibit potential as materials for applications involving blue light emission diodes (LEDs).

### Estimation of radiation defensive parameters via Phy – X/PSD

#### Mass attenuation coefficient (µ/ρ)

**Fig. 7:**
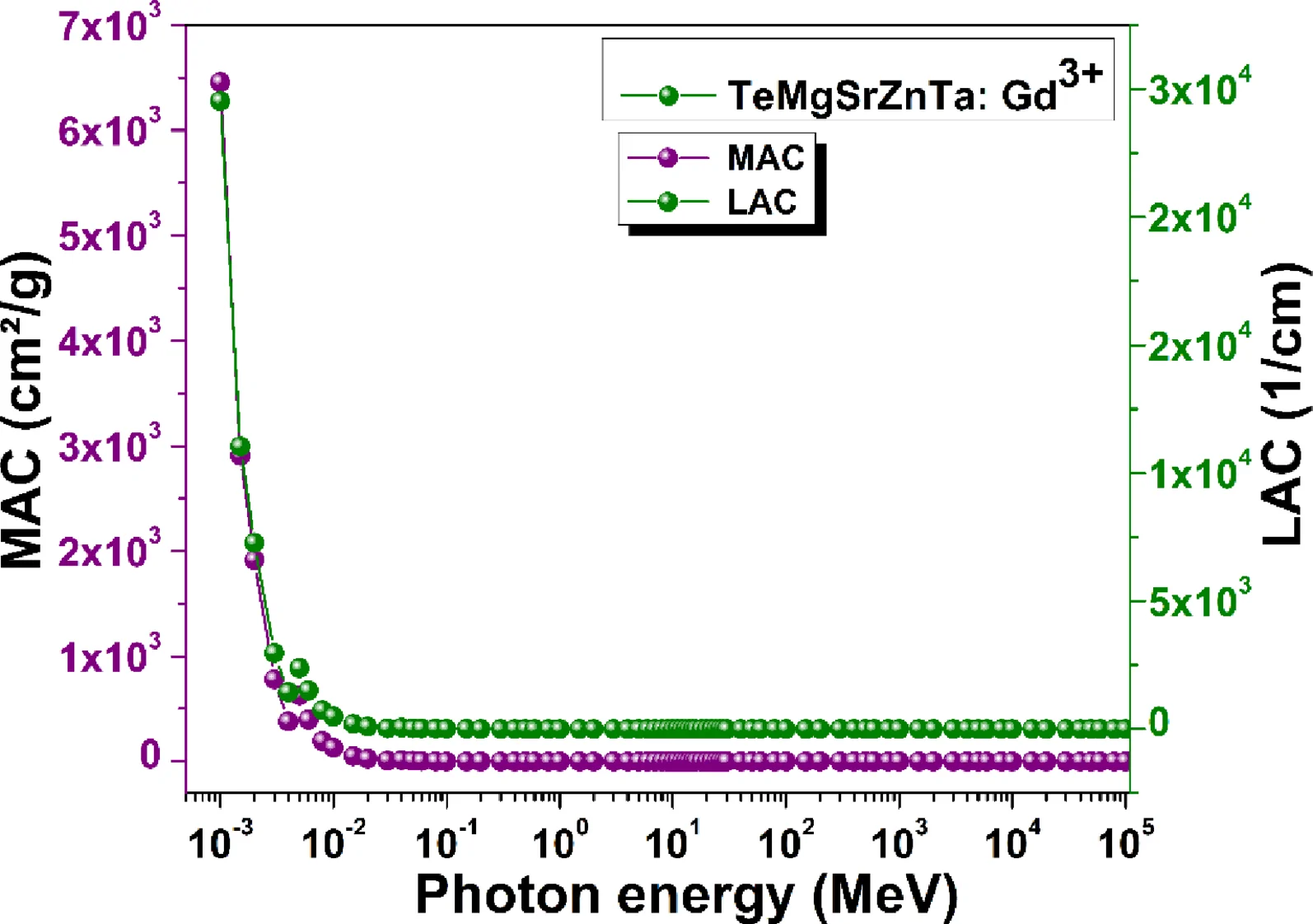
MAC & LAC Vs Photon energy for TeMgSrTa: Gd^3+^ glass

The mass attenuation coefficient (MAC, µ/ρ) of the TeMgSrTa³⁺ glass was calculated theoretically using the Phy-X/PSD software over a photon energy range of 0.001–15 MeV, as shown in Fig. 7. The results indicate that the glass exhibits significantly higher attenuation at lower photon energies, primarily due to the presence of Gd³⁺ ions, which enhance the probability of photon interaction. As the photon energy increases, the attenuation capability gradually decreases, resulting in only a marginal influence of Gd³⁺ at intermediate and high energies. This energy-dependent behavior is governed by the dominant photon interaction mechanisms. At low energies, the photoelectric effect predominates and accounts for the strong attenuation by complete absorption of photon energy. In the intermediate energy region, Compton scattering becomes the principal interaction process, whereas pair production dominates at higher photon energies, leading to the observed variation in the attenuation coefficients across the investigated energy range.

Thus, in the graphical representation shows, the µ/ρ measurements are forcefully reduced as the energy rises [5]. Furthermore, the attenuation converts linear and progressively constant due to the initial photons’ partial scattering in both PP and Compton scattering modes. The MAC (µ/ρ) and LAC of the Gd^3+^ ion doped TeMgSrTa glass lies between 6455.66 and 0.07543 cm²/g and 24531.45–0.28663 cm ^–1^ at 0.001–100,000 MeV. One possible explanation is that the cross-sections for PE, CS, and PP phases depend on Z4, Z, and Z_2_, respectively. However, the photoelectric was discovered to be a significant interaction. The absorption by photoelectric around the K – shaped absorption edge of Gadolinium ion (Z = 64) caused a sharp rise in the µ/ρ outcomes at an energy of 0.05 MeV [52]. The linear attenuation coefficient (LAC) is an efficient protecting characteristic that can be calculated from MAC estimations and shown in Fig. 7, against photon energy ranging from 0.015 to 15 MeV. LAC values follow the similar tendency as MAC outcomes, with Gd^3+^ doped TeMgSrTa glass exhibiting the greatest linear attenuation coefficient values [3].

#### HVL, TVL and MFP

**Fig. 8:**
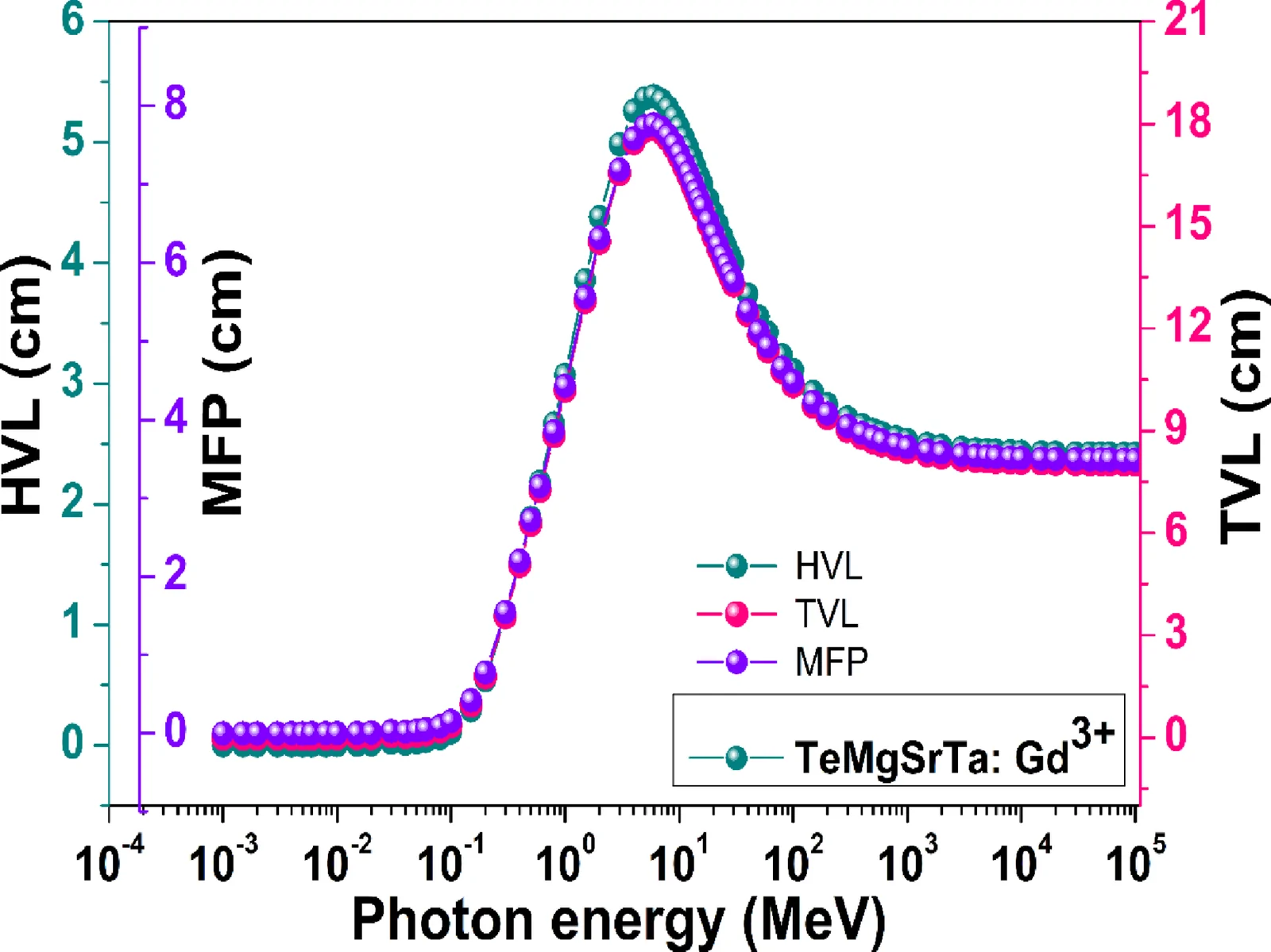
Half, 10th – value layer & MFP Vs photon energy of TeMgSrTa: Gd^3+^ glass

The half-value layer (HVL) is defined as the thickness of a material required to reduce the intensity of incident gamma radiation to one-half of its initial value, whereas the tenth-value layer (TVL) represents the thickness needed to decrease the radiation intensity to one-tenth. The variation of HVL and TVL with photon energy for the TeMgSrTa³⁺ glass is presented in Fig. 8a, b. Another important shielding parameter is the mean free path (MFP), which represents the average distance travelled by a photon within the material before undergoing an interaction. Lower values of HVL, TVL, and MFP indicate superior radiation shielding performance, as they reflect a greater probability of photon attenuation within a shorter penetration distance. Therefore, these parameters provide a reliable assessment of the effectiveness of the developed glass for gamma-ray and neutron shielding applications [35]. TeMgSrTa: Gd^3+^ glass showed essentially constant HVL, TVL and MFP, values up to 0.1 MeV, then quickly increased with increasing photon energy up to 6 MeV, after which the parameter values somewhat decreased.

The variation of the HVL, TVL, and MFP with photon energy demonstrates that these parameters increase gradually as the photon energy increases, reaching maximum values of 5.38 cm, 17.88 cm, and 7.76 cm, respectively, at 6 MeV. The low values observed at lower photon energies are primarily attributed to the dominance of the photoelectric absorption process, which results in efficient photon attenuation. As the photon energy increases, Compton scattering and pair production become the predominant interaction mechanisms, leading to a gradual increase in the shielding parameters. The high density and corresponding mass attenuation coefficient of the TeMgSrTa³⁺ glass contribute to its low HVL, TVL, and MFP values, indicating excellent radiation shielding efficiency. Furthermore, the shielding performance of the developed glass was compared with several commercially available shielding materials used in medical applications. The results demonstrate that the present glass exhibits superior attenuation performance compared with RS360 and RS253-G18 shielding glasses and shows performance comparable to RS520, confirming its suitability for advanced radiation protection applications. ^54^. The prepared glasses are more suited to be employed as a substitute defending material in medical field utilizations, such as protecting glass windows or directly protecting people undergoing diagnostic and interventional X-ray procedures [54].

#### Experimental measurements of LAC, MAC, HVL, TVL and MFP

The experimental and theoretical photon attenuation parameters of the developed glass are summarized in Table 1. The linear attenuation coefficient (LAC) decreases from 6.425 cm⁻¹ at 0.10 MeV to 0.367 cm⁻¹ at 0.50 MeV, indicating that the glass exhibits greater attenuation efficiency for low-energy photons. Similarly, the mass attenuation coefficient (MAC) decreases from 1.2356 cm² g⁻¹ to 0.0706 cm² g⁻¹ over the same energy range, reflecting the reduced probability of photon interaction at higher energies. In contrast, the mean free path (MFP) increases from 0.155 cm to 2.725 cm, demonstrating that high-energy photons travel longer distances before interacting with the material. A similar increasing trend is observed for the half-value layer (HVL) and tenth-value layer (TVL), which increase from 0.1079 cm to 0.3584 cm at 0.10 MeV to 1.8887 cm and 6.2741 cm at 0.50 MeV, respectively. These results confirm that thicker shielding is required for higher-energy gamma rays. Furthermore, the close agreement between the experimental and theoretical values of MAC, LAC, HVL, TVL, and MFP demonstrates the reliability of the attenuation calculations and validates the radiation shielding performance of the prepared glass.

**Table 1:** Experimental and theoretical values of various photon attenuation parameters

Photon attenuation coefficients		Photon energy (MeV)					
		0.1	0.15	0.2	0.3	0.4	0.5
LAC (cm⁻¹)	Experimental	6.162	2.011	1.145	0.599	0.42	0.326
	Theoretical	6.425	2.383	1.285	0.647	0.455	0.367
MAC (cm²/g)	Experimental	1.2356	0.4583	0.2471	0.1244	0.0875	0.0706
	Theoretical	1.691	0.627	0.338	0.170	0.120	0.097
HVL (cm)	Experimental	0.1079	0.2909	0.5394	1.0713	1.5234	1.8887
	Theoretical	0.108	0.291	0.540	1.072	1.525	1.887
TVL (cm)	Experimental	0.3584	0.9663	1.7919	3.5589	5.0606	6.2741
	Theoretical	0.358	0.966	1.792	3.561	5.065	6.268
MFP (cm)	Experimental	0.1556	0.4196	0.7782	1.5456	2.1978	2.7248
	Theoretical	0.156	0.420	0.778	1.546	2.200	2.722

Table 1 presents the experimental and theoretical photon attenuation parameters of the investigated glass in the energy range of 0.1–0.5 MeV, with both datasets showing good agreement. The experimental LAC and MAC decrease from 6.162 cm⁻¹ and 1.2356 cm² g⁻¹ at 0.1 MeV to 0.326 cm⁻¹ and 0.0706 cm² g⁻¹ at 0.5 MeV, owing to the reduced probability of photon interaction as the dominant attenuation mechanism shifts from the photoelectric effect at low energies to Compton scattering at intermediate energies. Conversely, the HVL, TVL, and MFP increase from 0.1079, 0.3584, and 0.1556 cm to 1.8887, 6.2741, and 2.7248 cm, respectively, indicating that higher-energy photons possess greater penetrating power and therefore require thicker shielding materials for effective attenuation. These findings demonstrate the superior shielding efficiency of the Gd³⁺-doped tellurite glass, particularly in the low-energy photon region.

### Effective FNRCS (Σ_R_)

The FNRCS (effective removal cross – sections: Σ_R_), which are nearly continuous with the neutron energy range 2 to 12 MeV, are a measure of the probability that a fast or separation energy neutron will endure a specific response per unit path length of transit over any protecting material. When inelastic scattering is no longer feasible at energies lower than the 1 st excited energy state of any nucleus in the glass materials, flexible scattering is the method left for removing fast neutrons. While the existence of low Z elements in glasses matrix which improves the FNRCS, a mix of lower and larger order of Z elements. Additionally, the estimated Σ_R_ = 0.101 cm^− 1^ outcomes for TeMgSrTa: Gd^3+^ glass is greater/lesser than that of standard and reported materials as follows: hematite serpentine (Σ_R_ = 0.097 cm^− 1^) and regular concrete, (Σ_R_ = 0.094 cm^− 1^) ^56^, B1–B6 (Σ_R_ = 0.105–0.092 cm^− 1^) and S1–S6 (Σ_R_ = 0.101–0.106) glasses [55], 80TeO_2_ – 20K_2_O (Σ_R_ = 0.092 cm^− 1^), 80TeO_2_ – 20ZnO (Σ_R_ = 0.100 cm^− 1^), and 80TeO_2_ – 20BaO (Σ_R_ = 0.101 cm^− 1^) glasses [56], S1 – S5 (Σ_R_ = 0.098–0.106 cm^− 1^) and P1–P6 (Σ_R_ = 0.093–0.108) glasses [57].

### Thermoluminescence behaviour

Thermoluminescence (TL) is a valuable characterization technique for investigating the trapping and recombination processes in luminescent materials. It provides important information on the nature, distribution, and depth of defect or trap levels present within the glass matrix. A thermoluminescence glow curve represents the variation of light emission intensity as a function of temperature during controlled heating. The position, shape, and intensity of the glow peaks are directly associated with the concentration and energy depth of trapping centers and recombination sites, thereby providing insight into the charge storage and luminescence behavior of the material [58–60]. The characteristics of a thermoluminescence glow curve, including the shape, peak temperature, and emission intensity, are strongly influenced by several factors, such as the type and concentration of rare-earth dopants, impurity levels, annealing conditions, and the structural nature of the material. Variations in these parameters alter the distribution and depth of trapping centers, thereby affecting the thermoluminescence response. Therefore, systematic investigation of the effects of dopant incorporation and thermal treatment on the glow curve is essential for optimizing the trapping characteristics and enhancing the dosimetric performance of advanced thermoluminescent materials [54, 61].

**Fig. 9:**
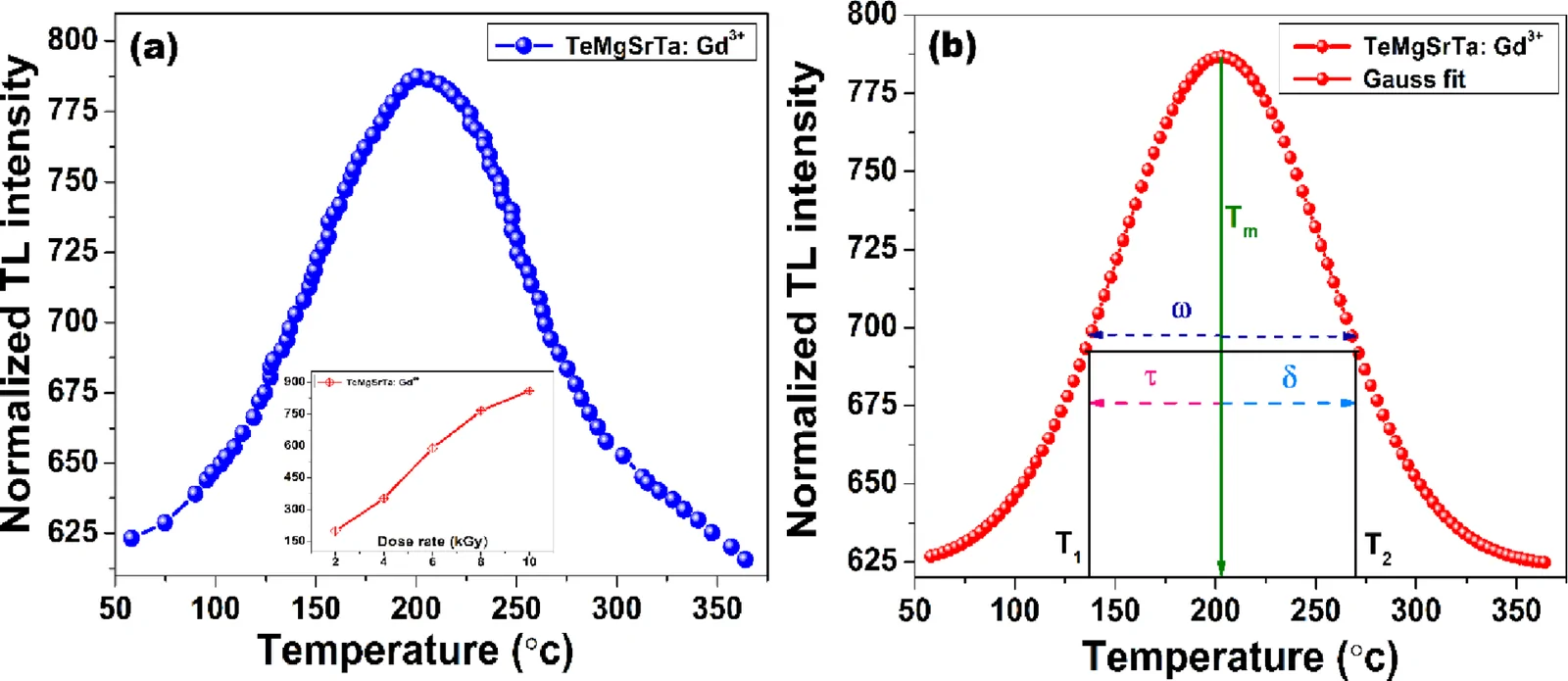
**a** Thermoluminescence glow curve Vs temperature, the inset plot of irradiation dose rate; **b** Gauss fit of glow curve for TeMgSrTa: Gd^3+^ glass

Thermoluminescence (TL) is a highly sensitive technique for investigating the trapping centers and defect states present in luminescent materials. In the present study, the thermoluminescence behavior of Gd³⁺-doped TeMgSrTa tellurite glass was examined to evaluate the trapping characteristics and structural modifications induced by rare-earth incorporation. Tellurite glasses are well known for their high refractive index, excellent infrared transparency, and good thermal stability, making them suitable host matrices for rare-earth ions. The incorporation of Gd³⁺ into the TeMgSrTa glass network introduces localized structural defects and trap levels that facilitate charge carrier trapping during gamma irradiation, thereby enhancing the thermoluminescent response and demonstrating the potential of the prepared glass for radiation dosimetry applications [62]. Figure 9a-b, illustrates the process utilized to extract the numerous kinetic parameters from the glow humps of thermoluminescent curve and gauss fit is estimated on the TeMgSrTa: Gd^3+^ glass samples. The gamma ray irradiation dose rate verses TL intensity of irradiated glass was inset in the Fig. 9a.

Following gamma irradiation and controlled heating, the TeMgSrTa³⁺ glass exhibited a well-defined thermoluminescence glow peak at 203.9 °C, corresponding to the maximum TL emission intensity. This peak represents the thermal release of trapped charge carriers, followed by their recombination at luminescence centers, resulting in light emission. The distinct glow peak indicates the presence of stable and well-defined trapping centers introduced by the incorporation of Gd³⁺ ions into the glass network. The kinetic parameters derived from the glow curve further characterize the trap distribution. The low-temperature half-width (τ) and high-temperature half-width (δ) were determined to be 70.6 °C and 66.86 °C, respectively, while the full width at half maximum (ω) was calculated as 133.29 °C. These parameters provide valuable information on the thermal stability and energy distribution of the trapping centers, confirming the favorable trapping characteristics of the prepared glass for thermoluminescent dosimetry applications.

The symmetry factor (µ_g_), which is the ratio of δ to ω, was calculated as 0.5. This value suggests that the thermoluminescence glow hump follows 1 st order kinetics, implying that the recombination of charged carriers arises without significant retrapping. In first-order kinetics, once the charged carriers are thermally unrestricted from traps, they immediately recombine with luminescent centers without being captured again by other traps. This behavior simplifies the interpretation of the glow curve and affirms the presence of energetically isolated and structurally stable traps introduced by Gd³⁺ doping. The near-symmetric shape of the glow peak also suggests a uniform defect environment and minimal interference from overlapping peaks. Utilizing the peak shape method applicable for 1 st order kinetics, the activation energy (Eʹ_α_) of the trapping center was estimated by Chen’s relation [28, 63], and yielded an activation energy of approximately 0.294 eV.

The calculated trap depth indicates that a moderate amount of thermal energy is required to release the trapped charge carriers, confirming the presence of thermally stable trapping centers. Such stable traps are advantageous for thermoluminescent dosimetry because they ensure good signal retention over extended periods. The frequency factor (s) was determined to be 1.12 × 10⁶ s⁻¹, which falls within the typical range reported for efficient thermoluminescent materials and reflects a favorable charge carrier release mechanism. The incorporation of Gd³⁺ ions, with their characteristic 4f⁷ electronic configuration, modifies the local glass structure by introducing lattice distortions and defect sites, including non-bridging oxygen atoms, which act as effective trapping centers. Consequently, the well-resolved glow peak, favorable kinetic parameters, and high thermal stability demonstrate that the TeMgSrTa³⁺ glass is a promising material for thermoluminescent dosimetry, radiation monitoring, and high-temperature sensing applications.

In conclusion, the thermoluminescence analysis of TeMgSrTa: Gd^3+^ glass reveals significant information about the trap depth and recombination kinetics. The glow peak at 203.9 °C, combined with a symmetry factor of 0.5 and an activation energy of 0.294 eV, confirms first-order kinetic behavior and the presence of stable, well-defined trap centers. These findings highlight the potential of Gd³⁺ ions in tailoring the luminescent and structural properties of tellurite glasses, enabling their application in devices requiring stable and reliable TL performance.

### Electron paramagnetic resonance (EPR) spectroscopy

**Fig. 10:**
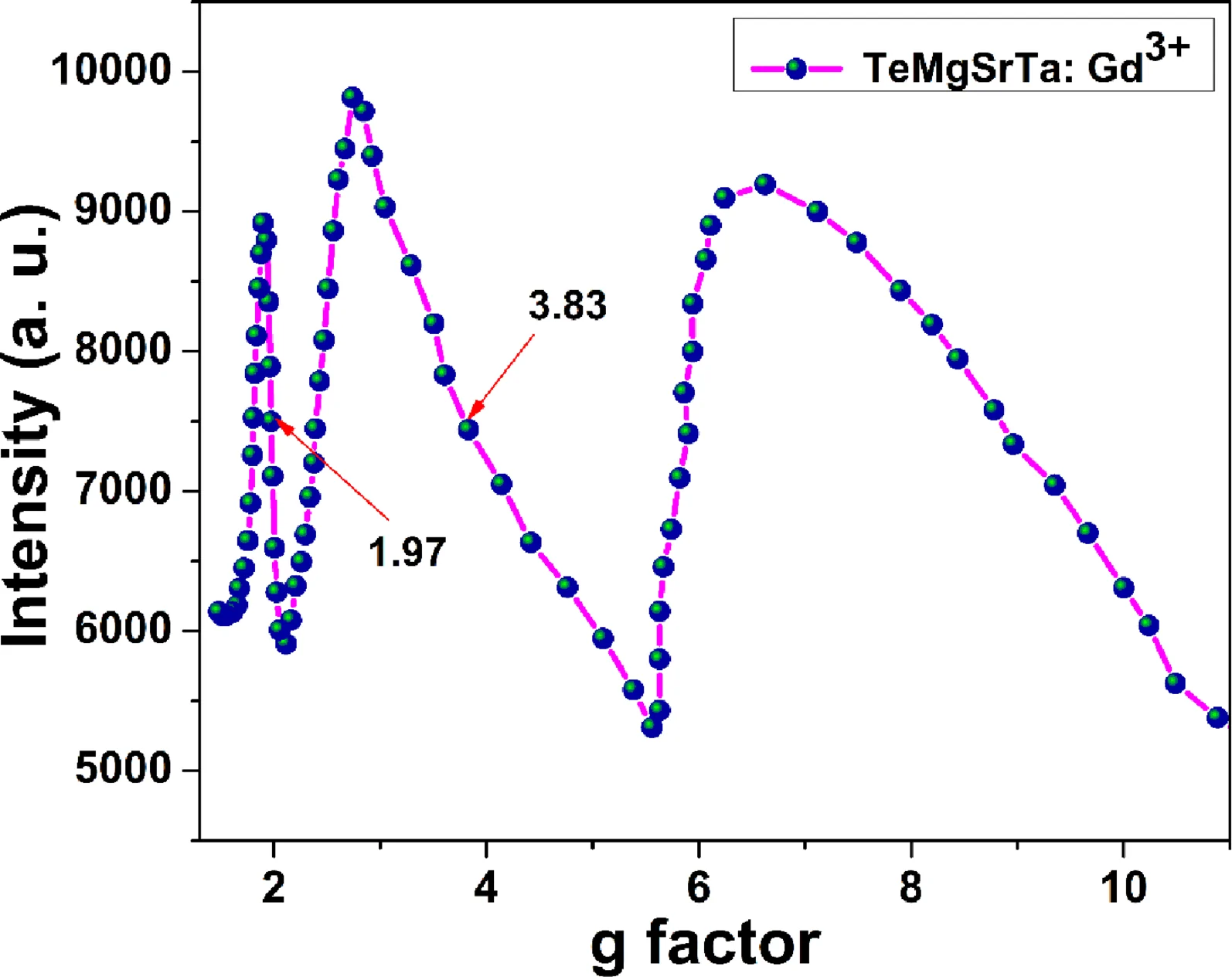
EPR spectrum Vs g – factor of TeMgSrTa: Gd^3+^ glass

Electron paramagnetic resonance (EPR) spectroscopy is a powerful technique for investigating the local environment of paramagnetic ions in glass materials. In the present study, EPR analysis was employed to examine the oxidation state, coordination environment, and local symmetry of Gd³⁺ ions incorporated into the TeMgSrTa glass network. Owing to its half-filled 4f⁷ electronic configuration (S = 7/2), Gd³⁺ possesses seven unpaired electrons, producing a strong and well-resolved EPR signal that is highly sensitive to its surrounding ligand field. The EPR spectrum of the TeMgSrTa³⁺ glass, recorded at room temperature in the X-band region, is presented in Fig. 10. Three distinct resonance signals with effective g-values of approximately 1.98, 3.60, and 9.0 were observed, indicating the presence of Gd³⁺ ions in different local coordination environments within the amorphous glass matrix. These resonance features reflect variations in the crystal field symmetry and magnetic interactions surrounding the Gd³⁺ ions, confirming their successful incorporation into the tellurite glass network [64].

The prominent resonance signal observed at g ≈ 1.98 is attributed to transitions between the Zeeman sublevels of Gd³⁺ ions in the high-field region. This signal indicates that a considerable fraction of the Gd³⁺ ions occupy relatively symmetric coordination sites with minimal structural distortion within the glass network. The sharp and well-resolved resonance suggests a weak crystal field surrounding the Gd³⁺ ions, consistent with octahedral or near-octahedral local coordination. Because Gd³⁺ possesses a half-filled 4f⁷ electronic configuration with an S-state ground state, its magnetic moment is nearly isotropic, resulting in a strong EPR response in environments with low ligand-field asymmetry. The observed resonance therefore confirms the stable incorporation of Gd³⁺ ions into the amorphous tellurite glass matrix while preserving a relatively symmetric local coordination environment [65].

In contrast, the g ≈ 3.83 resonance signal reflects the presence of Gd³⁺ ions in more distorted or lower-symmetry environments. This intermediate-field signal is typically attributed to transitions involving the |±1/2⟩ ↔ |±3/2⟩ sublevels, which are split due to the zero-field splitting (ZFS) and crystal field effects. The emergence of this g-value in the EPR spectrum indicates the existence of asymmetrical ligand fields, possibly arising from structural disorder, non-bridging oxygen atoms, or defects within the tellurite matrix. These distortions influence the local field experienced by the Gd³⁺ ions, leading to a broader resonance feature with reduced intensity compared to the principal g = 1.98 peak.

The third and most remarkable resonance signal appears at g ≈ 9, a region typically associated with highly distorted environments and strong zero-field splitting. This low-field signal can be ascribed to transitions between the |±7/2⟩ ↔ |±5/2⟩ levels, which become partially allowed under high ZFS conditions. Such a peak often indicates the presence of Gd³⁺ ions trapped in sites with substantial asymmetry, possibly at interstitial or defect-related locations within the glass network. The detection of this signal confirms the heterogeneous nature of the local surroundings of Gd³⁺ in the TeMgSrTa glass and suggests that multiple distinct coordination geometries coexist within the amorphous structure [66].

The coexistence of these three distinct g-values suggests a distribution of Gd³⁺ sites within the glass, reflecting the non – crystalline/amorphous structure of the tellurite network. Tellurite glasses, composed primarily of TeO₄ trigonal bipyramids and TeO₃+1 units, possess a flexible structure that can accommodate dopant ions in a variety of environments. The substitution of Te⁴⁺ by Gd³⁺ introduces charge imbalance and local distortions, promoting the formation of both regular and irregular coordination centers. The interaction of Gd³⁺ with non-bridging oxygens and structural defects further contributes to the observed g-value distribution.

The presence of multiple g-values in the EPR spectrum indicates that Gd³⁺ ions occupy more than one local coordination environment within the amorphous glass matrix, reflecting the magnetic anisotropy of the system. Variations in the g-factors arise from differences in the local ligand field symmetry and the structural disorder surrounding the Gd³⁺ ions. The deviation of the observed g-values from the free-electron value (g = 2.0023) is attributed to the combined effects of spin–orbit coupling and crystal field interactions, which are characteristic of heavy-metal oxide glasses such as tellurite systems. Furthermore, the narrow resonance observed at g ≈ 1.98 indicates relatively uniform coordination sites with limited spin relaxation, whereas the broader signals at g ≈ 3.60 and g ≈ 9.0 suggest the presence of more distorted local environments and stronger magnetic interactions, possibly arising from dipole–dipole coupling or weak clustering of Gd³⁺ ions within the glass network [67]. However, the absence of line splitting or significant broadening suggests that Gd³⁺ ions are well-dispersed in the matrix and not prone to agglomeration, preserving the amorphous integrity of the host. Table 2 compares the multifunctional characteristics of the developed Gd³⁺-activated tellurite glass with recently reported rare-earth-doped glass systems. Table 2 compares the radiation shielding characteristics of the present glass with recently reported rare-earth-doped glass systems (Table 3).

**Table 2:** Comparison of the present Gd³⁺-activated tellurite glass with recently reported rare-earth-doped glass systems

S.No	Glass composition	Eg (eV)	Main PL emission (nm)	Density (g cm⁻³)	MAC at 0.1 MeV (cm² g⁻¹)	ΣR (cm⁻¹)	Reference
1	Dy₂O₃–borate–tellurite–germanate	3.82	482, 575	4.86	5.12	0.097	Kaky et al. [52]
2	Tm³⁺-doped GeO₂–MgO–TeO₂–B₂O₃	3.95	455, 478	4.58	4.94	0.095	Abdel Wahab et al. [26]
3	Gd/Yb/Tm-doped GTB glass	3.88	475–650	5.12	5.31	0.099	Mhareb et al. [53]
4	Pr₂O₃-modified tellurite glass	3.69	488, 603	5.43	5.47	0.101	Gaafar et al. [33]
5	Fluoride–oxide glass	4.12	—	4.31	4.68	0.093	Krishnan et al. [29]
6	Nd³⁺/TiO₂ tellurite glass	3.76	882, 1062	5.24	—	—	Mustafa et al. [32]
7	Gd³⁺ aluminosilicate glass	4.48	UV absorption	2.73	—	—	Jucá et al. [43]
	Gd³⁺ lithium aluminium borate	4.18	312	2.84	—	—	Nagaraju et al. [50]
Present work	65TeO₂–15MgO–9SrO–5ZnO–5Ta₂O₅–1Gd₂O₃	4.29	313	3.8	6455.66*	0.101	Present work

Comparison of radiation

**Table 3:** Shielding parameters of the present glass with recently reported rare-earth-doped glass systems

S.No	Glass composition	Photon energy (MeV)	MAC (cm² g⁻¹)	LAC (cm⁻¹)	HVL (cm)	MFP (cm)	Fast neutron removal cross-section, ΣR (cm⁻¹)	Reference
1	Dy₂O₃–B₂O₃–TeO₂–GeO₂ glass	0.1	5.12	24.9	0.028	0.04	0.097	Kaky et al. [52]
2	Tm₂O₃–GeO₂–MgO–TeO₂–B₂O₃ glass	0.1	4.94	22.6	0.031	0.044	0.095	Abdel Wahab et al. [26]
3	Gd/Yb/Tm-doped GTB (GeO₂–TeO₂–B₂O₃) glass	0.1	5.31	27.2	0.026	0.037	0.099	Mhareb et al. [53]
4	Pr₂O₃-modified tellurite glass	0.1	5.47	29.7	0.023	0.034	0.101	Gaafar et al. [33]
5	Lead-free alkali-modified fluoride–oxide glass	0.1	4.68	20.2	0.034	0.05	0.093	Krishnan et al. [29]
6	TiO₂–Bi₂O₃–B₂O₃–TeO₂ glass	0.1	5.38	28.5	0.024	0.035	0.1	Al-Ghamdi et al. [35]
7	NiO-doped boron–tellurite bioactive glass	0.1	5.2	26.1	0.027	0.038	0.098	Kavun [54]
8	65TeO₂–15MgO–9SrO–5ZnO–5Ta₂O₅–1Gd₂O₃	0.001–100,000	6455.66–0.07543	24531.45–0.28663	2.82 × 10⁻⁵–2.42	4.08 × 10⁻⁵–3.49	0.101	Present work

### Thermal analysis

**Fig. 11:**
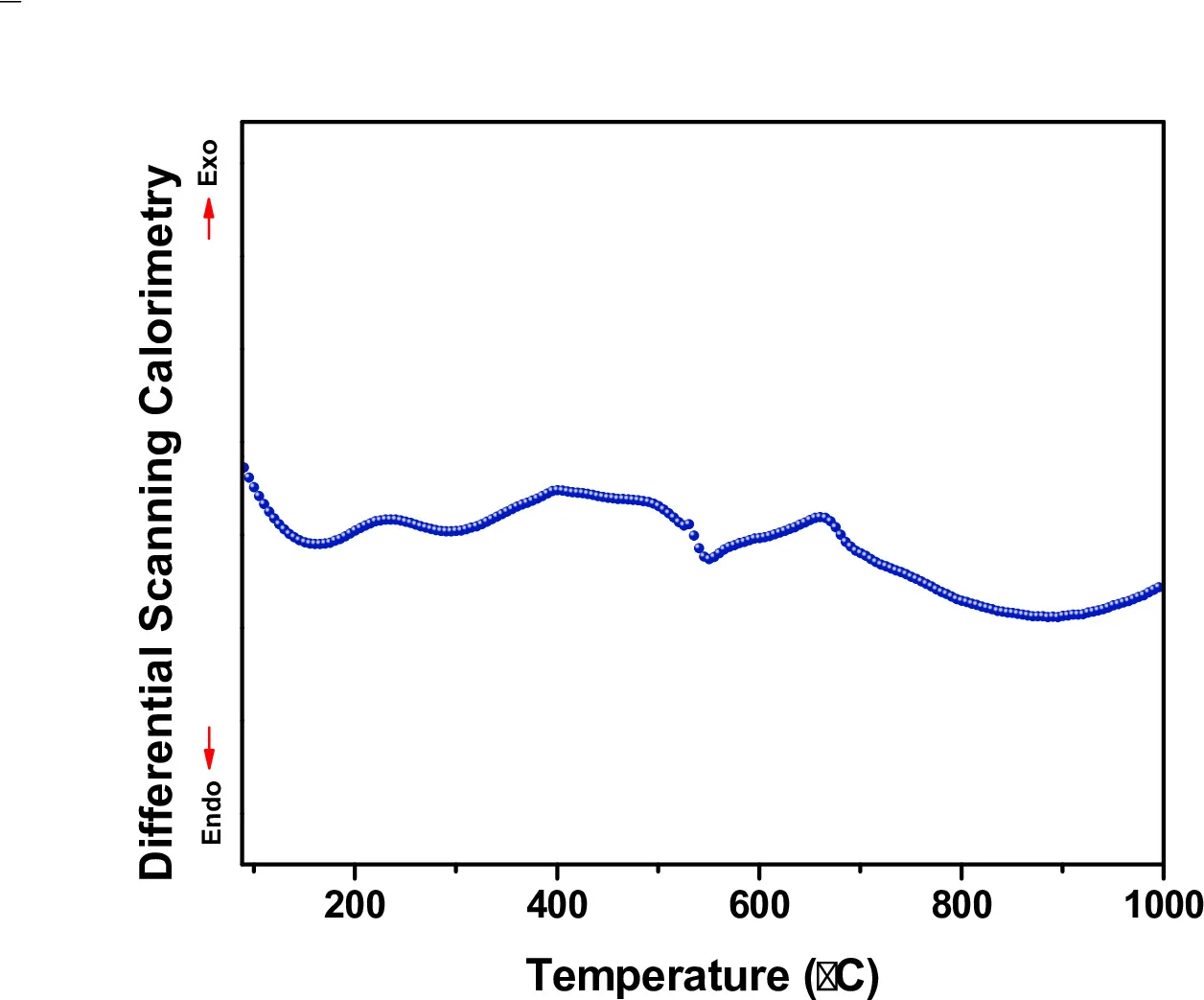
DSC curve of prepared glass

The thermal characteristics of the synthesized prepared glass were investigated by differential scanning calorimetry (DSC) using a NETZSCH DSC 404 F3 Pegasus Differential Scanning Calorimeter. Approximately 15 mg of finely powdered glass was sealed in a high-purity alumina crucible, with an identical empty crucible serving as the reference. Before the measurements, the instrument was calibrated using standard reference materials to ensure accurate temperature and heat-flow measurements. The experiment was carried out under a continuous flow of high-purity nitrogen **(**50 mL min⁻¹**)** to eliminate atmospheric interference during heating. The sample was heated continuously from 30 °C to 1000 °C at a constant heating rate of 10 °C min⁻¹, and the heat-flow response was recorded throughout the entire heating cycle. The thermal profile was interpreted over different temperature regions to identify the characteristic transitions of the glass. Figure 11 represents DSC curve of fabricated glass.

The region between 30 and 350 °C was examined for thermal stability at low temperatures, while the interval from 350 to 450 °C was carefully monitored for the onset of the glass transition (Tg). The temperature range of 450–550 °C was analyzed to determine the onset of crystallization **(**Tx) and the crystallization peak (Tc), whereas the 550–700 °C region was evaluated for any subsequent thermal events associated with structural rearrangement or progressive crystallization. The values of Tg, Tx, and Tc were determined directly from the DSC thermogram, and the thermal stability parameter (ΔT = Tx − Tg) was calculated to assess the resistance of the glass against devitrification during heat treatment. These thermal parameters were subsequently used to establish suitable processing conditions for further structural, optical, and luminescence investigations of the TeMgSrTa: Gd³⁺ glass.

## Conclusion

The successful fabrication of Gd³⁺-doped tellurite glass with the composition 65TeO₂–15MgO–9SrO–5ZnO–5Ta₂O₅–1Gd₂O₃ demonstrates the feasibility of developing a multifunctional glass that integrates structural stability, optical performance, radiation shielding, and dosimetric characteristics within a single matrix. X-ray diffraction confirmed the amorphous nature of the prepared glass, while FTIR analysis verified the coexistence of TeO₄ and TeO₃ structural units, indicating that the incorporation of Ta₂O₅ and Gd³⁺ effectively modified the tellurite network. UV–Visible spectroscopy revealed high optical transparency with an optical bandgap of 4.29 eV, suggesting good suitability for ultraviolet photonic applications. Photoluminescence measurements exhibited the characteristic Gd³⁺ emission, and the corresponding CIE 1931 chromaticity coordinates confirmed efficient blue–violet emission, highlighting the potential of the glass for ultraviolet and solid-state lighting devices. The radiation shielding investigation demonstrated excellent attenuation performance over a broad photon energy range (0.001–100000 MeV), with the mass attenuation coefficient (MAC) and linear attenuation coefficient (LAC) varying from 6455.66 to 0.07543 cm² g⁻¹ and from 24531.45 to 0.28663 cm⁻¹, respectively. The associated half-value layer, tenth-value layer, and mean free path further confirmed the superior photon shielding efficiency, while the fast neutron removal cross-section (ΣR = 0.101 cm⁻¹) indicated effective neutron attenuation. Thermoluminescence analysis produced well-resolved glow peaks with a frequency factor of 1.12 × 10⁶ s⁻¹ and favourable kinetic parameters, demonstrating the suitability of the glass for radiation dosimetry. Electron paramagnetic resonance spectroscopy revealed resonance signals with g-values of 1.98, 3.6, and 9.0, providing valuable information on the valence state, coordination environment, and local ligand field symmetry of Gd³⁺ ions. The novelty of this investigation lies in the comprehensive correlation established between the structural evolution of the tellurite network and its optical, luminescent, dosimetric, EPR, and radiation shielding responses, thereby demonstrating that the developed TeMgSrTa³⁺ glass is a promising multifunctional material for advanced photonic devices, radiation protection systems, and thermoluminescent dosimeters.

## Data Availability

The datasets used and/or analysed during the current study are available from the corresponding author on reasonable request.
